# The Interaction between Vector Life History and Short Vector Life in Vector-Borne Disease Transmission and Control

**DOI:** 10.1371/journal.pcbi.1004837

**Published:** 2016-04-29

**Authors:** Samuel P. C. Brand, Kat S. Rock, Matt J. Keeling

**Affiliations:** 1 School of Life Sciences, University of Warwick, Coventry, United Kingdom; 2 WIDER (Warwick Infectious Disease Epidemiology Research) Centre, University of Warwick, Coventry, United Kingdom; 3 Mathematics Institute, University of Warwick, Coventry, United Kingdom; Universite de Neuchatel Institut de Chimie, SWITZERLAND

## Abstract

Epidemiological modelling has a vital role to play in policy planning and prediction for the control of vectors, and hence the subsequent control of vector-borne diseases. To decide between competing policies requires models that can generate accurate predictions, which in turn requires accurate knowledge of vector natural histories. Here we highlight the importance of the distribution of times between life-history events, using short-lived midge species as an example. In particular we focus on the distribution of the extrinsic incubation period (EIP) which determines the time between infection and becoming infectious, and the distribution of the length of the gonotrophic cycle which determines the time between successful bites. We show how different assumptions for these periods can radically change the basic reproductive ratio (*R*_0_) of an infection and additionally the impact of vector control on the infection. These findings highlight the need for detailed entomological data, based on laboratory experiments and field data, to correctly construct the next-generation of policy-informing models.

## Author Summary

The basic reproductive ratio (*R*_0_) is a crucial measure of transmission intensity, lying at the interface between mathematical modelling and policy decision making. If control measures can induce a situation where *R*_0_ ≤ 1 for a sustained period of time then the pathogen must be eradicated. For diseases spread by short-lived insect vectors a modeller can not calculate *R*_0_ without addressing questions of chance such as, “What percentage of vectors will survive their extrinsic incubation period (EIP) to become infectious?”. Classical Ross-Macdonald theory provides answers for the modeller by making certain concrete assumptions, such as a fixed length EIP and exponentially distributed times between vector blood-meals. Using bluetongue virus spread by biting midges as an exemplar we demonstrate that biologically plausible alterations to the classical assumptions can significantly change the modeller’s prediction of *R*_0_ with both serious over-estimation and under-estimation being possible. The important modelling/control question, “How does *R*_0_ respond to increased vector per-capita mortality?”, is also found to depend strongly on details of the vector life-cycle expressed in the language of probability distributions.

## Introduction

The language of probabilities and chance entered mathematical epidemiology, then dubbed pathometry, almost from its foundations [1]. However, the common use, and huge success, of deterministic models for disease dynamics (ODEs) have made certain probabilistic assumptions very popular in the literature; for example the implicit assumption that the time between various epidemiologically important events is exponentially distributed, which follows from assuming constant per capita rates of change. For vector-borne diseases (VBDs) the ODE-based approach dominates, originating with Ross’ modelling of malaria [2]. In the 1940s and ‘50s Macdonald and Garrett-Jones, amongst others, made substantial contributions to the mathematical theory of VBDs [3, 4], including the fundamental appreciation that only a fraction of inoculated vectors will survive the extrinsic incubation period (sometimes called sporogony) of a disease to become actively infectious. The probability of surviving the extrinsic incubation period (EIP) has been most conveniently expressed by assuming that the EIP duration is fixed [3, 4]. The epidemiologically important factors in a vector’s life history are its life duration, how many times it bites in its life and how many of those bites are infectious. Variation between vector life histories can be modelled as probabilistic; the difference in outcomes between vectors are modelled as being due to identically distributed chance factors rather than intrinsic variation in vector fitness. This requires estimation of the underlying random distributions governing vector life histories. The popularity in the modelling literature of implicitly assuming that relevant distributions are either exponential or fixed length is often due to the popularity of ODE models and mathematical convenience rather than biological motivation.

Ross-Macdonald theory has remained very popular amongst mathematical modellers of VBDs. A century after Ross’ original mathematical analysis, reviews of the modelling literature reveal that modern analysis still typically uses the majority of the core set of assumptions familiar to Ross, Macdonald and Garrett-Jones [5]. This has led to speculation that the entire field could have become ‘canalised’ implying that there exist equally fruitful avenues of research that are being under-explored due to the popularity of the Ross-Macdonald approach [6]. However, Ross-Macdonald theory is essentially an attempt to think clearly and quantitatively about the vector life events that must occur in the host-to-vector-to-host transmission cycle of VBDs. In particular their theory allows the integration of important entomological information [the vector biting rate (*α*), vector mortality rate (*μ*) and the vector population density relative to hosts (*M*)] with epidemiological data [the vector competence for the given pathogen (*V*) and the extrinsic incubation period, or inverse incubation rate, (*σ*^−1^)] into epidemic metrics for VBDs that give a prediction for transmission intensity. A popular metric is the vectorial capacity (*C*), first proposed by Garrett-Jones [4], which measures the expected number of future infected hosts due to a single infectious host per day [7].

If we assume the host has mean infectious duration 1/*γ*, which includes host mortality and possible excess mortality due to disease, and additionally assume that the host is certain to survive its incubation period, then combining vectorial capacity with the mean duration of host infectiousness gives the classical reproductive ratio for the VBD as

$$R_{0}^{c}=C/\gamma =\left(\frac{MV\alpha^{2}}{\gamma \mu }\right)\exp\left(-\mu /\sigma \right).$$ (1)

We use the notation $R_{0}^{c}$ for the classical Macdonald reproductive ratio for a VBD. However, in relationship to other common definitions of *R*_0_ (e.g. [8]) it could equally be described as $R_{0}^{2}$ as the square of the geometric mean of two infectious generations: vectors and hosts. $R_{0}^{c}$ is a threshold quantity for the epidemic; that is if control efforts can induce a situation where $R_{0}^{c}\leq 1$ for a sustained period the VBD must be eradicated [8]. On the other hand, recent analysis [9, 10] has suggested that the reproductive ratio for the midge-borne viral disease bluetongue virus (BTV) spreading between a single host population and a single midge species can be expressed as,

$$R_{0}=\left(\frac{MV\alpha^{2}}{\gamma \mu }\right)\left(\frac{\sigma }{\sigma +\mu }\right).$$ (2)

The discrepancy between the two formula is due to the interaction between the lifetime of the vector, modelled as an exponential distribution, and the assumed distribution of the EIP. $R_{0}^{c}$ (eq 1) is derived from assuming a constant (fixed) duration EIP, whereas *R*_0_ (eq 2) is generated by assuming an exponential distribution as epitomised by ODE models. Despite both models assuming the same *average* EIP duration (1/*σ*) and vector life expectancy (1/*μ*), they predict different probabilities of an inoculated (female) vector surviving her EIP and therefore becoming actively infectious. When the incubation rate is much faster than the mortality rate (*σ* ≫ *μ*) the two *R*_0_ predictions become identical because the vector is very likely to survive its EIP and probabilistic details become irrelevant. This will not be true for vectors (such as midges and sandflies) that are short lived compared to their typical EIP duration.

The classic assumption that the lifetime of a vector is exponentially distributed is equivalent to assuming that it has a constant hazard rate of death. In this we follow Macdonald [11] and argue that due to predation, and other persistent environmental risks to the vector, exponentially distributed lifetimes are a reasonable model for many vectors. Constant hazard is probably a more accurate modelling choice for smaller arthropods (with less complex life-histories) such as biting midges or sandflies than compared to their larger cousins such as mosquitos and tsetse flies. Certain populations of the biting midge species *Culicoides sonorensis* have been found to have a lifetime distribution that closely matches exponential, even in a laboratory setting where predation pressures and other environmental risks are absent [12]. In addition, field estimates of mortality based on the proportion of parous vectors caught implicitly assume exponential lifetimes (e.g. [13]). For larger arthropods there is stronger evidence of senescence [14], however laboratory studies of this facet represent an upper estimate on the survivorship of arthropods in the natural setting.

In contrast, we would question the appropriateness of the exponential distribution as a good model for other biological durations significant to the epidemiology of VBDs. Vectors cannot become immediately infectious after successful inoculation with a VBD due to the time required for the pathogen to escape the mid-gut of the vector and spread to its salivary glands from where it can be transmitted to susceptible hosts; it is this biological process that defines the EIP for the vector. It does not seem plausible that this duration should be identical for each vector, as implied by Eq (1) but nor does it seem plausible that the EIP should be modelled as ceasing at a constant rate after inoculation, as implied by Eq (2). Moreover, vectors such as female biting midges and mosquitos generally only need to successfully feed once in their gonotrophic cycle in order to provide protein for their egg yolks as well as for sustenance. Even if there is a local abundance of suitable hosts and breeding sites, implying that the vector spends only a short period in seeking behaviour, the biting activity of the vector is still limited by the duration of oogenesis (egg production) and oviposition (egg laying). Since oogenesis is a complex multi-stage biological process, rather than a persistent ‘risk’, it is unlikely that the duration between bites by a female midge is exponentially distributed.

We introduce a flexible approach to modelling vector life histories as random events during an individual vector’s life. The random waiting time between bites on hosts is drawn from a general distribution, that does not have to be the standard exponential. The generalisation of the vector biting process to non-exponentially distributed gonotrophic cycle duration defines a renewal process, a standard theoretical model for processes of discrete events [15]. The EIP duration is also allowed to be chosen from an arbitrary positive distribution; the probability of a vector surviving its EIP duration can then be expressed analytically. Combining these two insights we develop a numerical procedure for calculating a generalised reproductive ratio ($R_{0}^{g}$), and also an analytic approximation (${\tilde{R}}_{0}^{g}$) based on the renewal process for biting rate of longer-lived vectors. Given the uncertainty in the forms of these distributions, it is convenient to introduce a dispersion scale, which interpolates between the fixed-duration, dispersion-zero, gonotrophic cycle assumption Eq (1) and the exponentially-distributed, dispersion-one, gonotrophic cycle assumption Eq (2). This approach does not capture all possible distributional choices, but does include the two most popular [eqs (1) and (2)]. We demonstrate that for short-lived vectors these changes have a significant effect on both predicted intensity of VBD transmission and the efficacy of control measures.

*Culicoides* genus biting midges spreading the multi-serotype *orbivirus* bluetongue virus (BTV) provide an ideal case study for the theory developed, due to the plethora of entomological and virological data available on the midge-BTV disease complex. BTV causes the economically important bluetongue disease amongst ruminants, both wild animals and commercial livestock, and is in particular associated with severe disease and increased mortality amongst sheep. BTV circulates persistently in Africa, North America, Australia, the Middle east, China, the Indian subcontinent and southern Europe wherever *culicoides* midge species are present. Moreover, the virus has demonstrated epizootic invasion capability into northern Europe (north of 50°N) [16]. In 2006 BTV invaded northern European herds of commercial ruminant livestock for the first time in record, and demonstrated the ability to overwinter before being controlled by mass vaccination campaigns of over 100 million animals in the subsequent years [17]. The generalised predictions from this paper show that the effort required to achieve eradication by host vaccination or insecticidal spraying can be substantially greater or lower than those offered by classical Ross-Macdonald theory, depending on the assumed underlying distributions. However, we show that whenever $R_{0}^{g}$ agrees with $R_{0}^{c}$, the classical approach overestimates the efficacy of reducing vector life expectancy with adulticidal spraying. As experimental knowledge about midge gonotrophic cycles and EIP increases and becomes more detailed [18, 19] there should be an concurrent effort from modellers of VBDs to integrate more of this detailed entomology into predictions of disease risk. The theory presented in this paper allows the full distributional information on the length of gonotrophic cycle and EIP to be integrated into a prediction of transmission intensity: this represents a significant development from the classical formulation for *R*_0_ [eq (1)] where only information about *averages* can be used to estimated disease risk.

## Methods

In this section we highlight two processes that combine to determine the reproductive ratio of vector-borne diseases: surviving the EIP and the timing of vector bites. In particular we distinguish between biting from the vector population in general and infectious bites from long-lived inoculated vectors.

### Model overview

**Fig 1:**
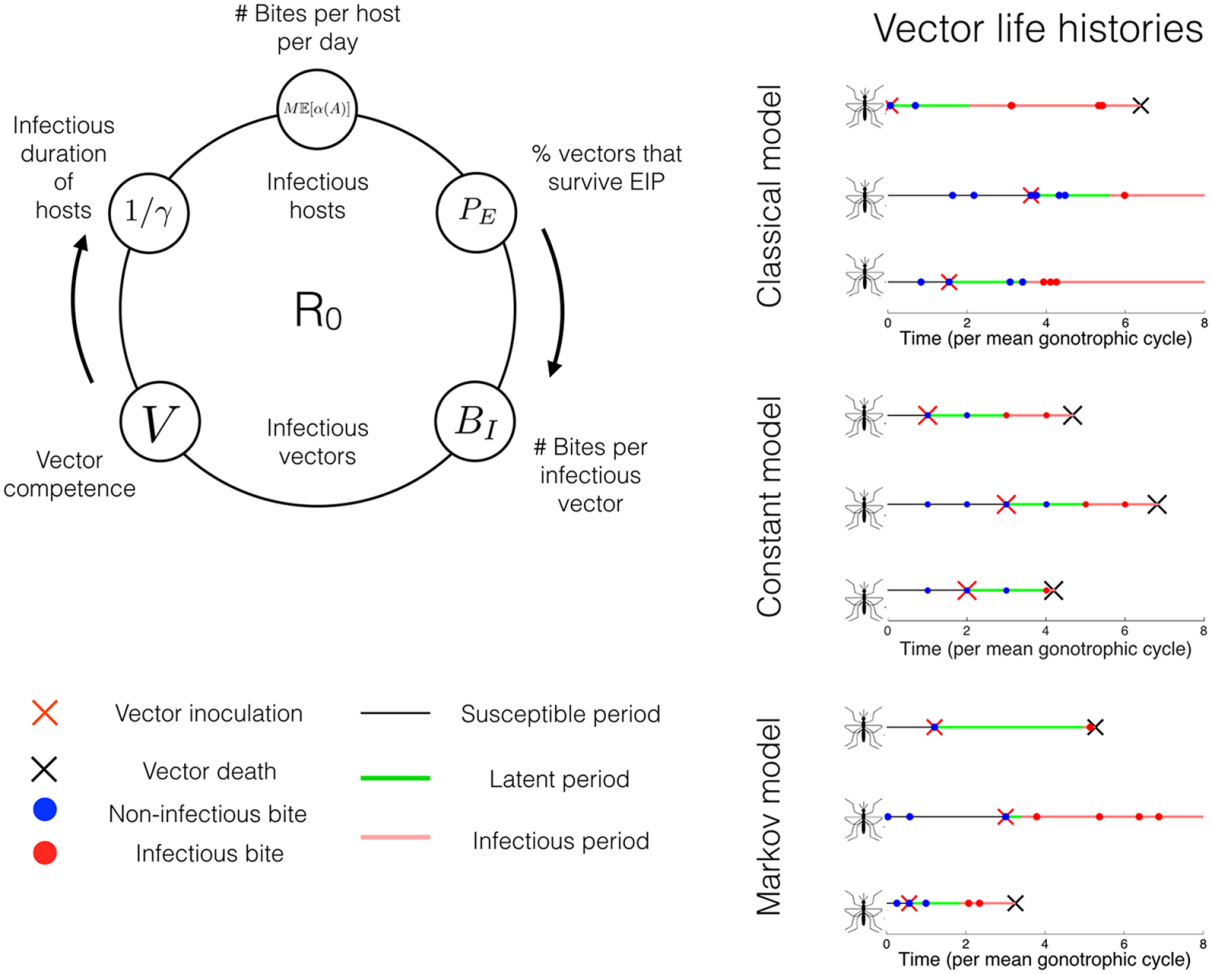
Reproductive ratio and vector life history examples. The generalised reproductive ratio, $R_{0}^{g}$, is derived by computing the expected number of vertebrate hosts infected through one complete generation of the parasite due to a single infected host. The average quantities underlying $R_{0}^{g}$ depend upon assumptions on the chance life events of individual vectors. Three example realisations of vector lives are given for three extremal modelling assumptions (*Right*). 1) The classical model. The vector waits for an exponentially distributed period between bites (circles), once inoculated with the pathogen the vector has a fixed latency period (green line) until the end of its EIP after which it makes infectious bites. 2) The constant model. Both the EIP duration and the time between bites are fixed. 3) The Markov model. Both the time between bites and the EIP duration are exponentially distributed. In each case the vector’s life ends with a fixed per-capita mortality rate.

The adult lives of the vectors are modelled as a series of independent and identically distributed (i.i.d) random waiting periods—the gonotrophic cycles—which we label *G*_*n*_ (*n* ≥ 0). These define the time between each successful bite until death, and death is assumed to occur at constant rate *μ* (Fig 1). This means that the lifetime biting process of the vector follows a renewal process [15] stopped at the end of the vector’s life. The starting point of the gonotrophic cycles for each vector is taken to be its emergence as an adult. If a vector is inoculated with the pathogen, then only bites after a random duration *E* (the EIP) are potentially infectious. The product of the probabilities of transmission from infectious host to vector and from infectious vector to host (the vector competence) is given as *V*. The classical assumption is that the *G*_*n*_ are exponentially distributed (*G* ∼ exp(*α*)) and *E* = 1/*σ* is constant. Here we drop these assumptions and generate a *generalised* reproductive ratio ($R_{0}^{g}$) for a single midge species spreading a pathogen amongst a single host species (Fig 1). Assuming that the average host infectious duration is 1/*γ*, $R_{0}^{g}$ expressed in explicitly probabilistic terms:

$$R_{0}^{g}=\frac{V}{\gamma }\times E[\text{rate of vector population bites per host}]\;\times E[\#\text{ bites after EIP by inoculated vector}].$$ (3)

The factorisation in eq (3) is appropriate because the future biting of a vector after its inoculation can be taken as independent of its past life-history. The probability of a vector surviving its EIP duration and the average biting rate over the vector population can be solved in terms of the moment generating functions (MGFs) of the random life-cycle distributions for the vector. The MGF for a random variable *X* is defined as,

$$\phi_{X}\left(\theta \right)=E\left[\exp\left\{-\theta X\right\}\right].$$ (4)

From an entomological modelling perspective the MGF evaluated at *θ* > 0 is the probability of a vector surviving a random period defined by *X* whilst undergoing constant mortality rate *θ*. The two MGFs we will use in our analysis are *ϕ*_*E*_, the MGF of the EIP duration, and *ϕ*_*G*_, the MGF of the gonotrophic cycle duration. Therefore the probability of a vector surviving its EIP,

$$P_{E}=\phi_{E}\left(\mu \right).$$ (5)

The classical assumption that the EIP is fixed at *σ*^−1^ for each inoculated vector in fact leads to the smallest estimate of *P*_*E*_ over *any* choice of distribution for the EIP with average duration *σ*^−1^ [eq (15)]; that is the classical estimate is almost certainly an over-estimate for the proportion of inoculated vectors that die before becoming actively infectious.

The average number of bites, or equivalently survived gonotrophic cycles, in the lifespan of a vector is,

$$E\left[\text{number of lifetime bites}\right]=\frac{\phi_{G}\left(\mu \right)}{1-\phi_{G}\left(\mu \right)}.$$ (6)

A traditional modelling assumption is that each vector bites according to a Poisson process at rate *α*(each gonotrophic cycle is exponentially distributed with a mean cycle duration of 1/*α*) giving a population biting rate per host of *Mα* where *M* is the vector-to-host population ratio. A convenient feature of modelling vector bites as a Poisson process is that the chance of the vector biting at any moment in time is completely independent of the vector age, *a*, since emerging as an adult. This is not true in general; it is biologically plausible that having bitten successfully a vector is less likely to bite shortly afterwards, even if the average time between bites remains 1/*α*. This could be experimentally confirmed by observing that gonotrophic cycles are under-dispersed compared to an exponential distribution. As a consequence vectors of different ages will have different chances of biting: vector age dependence in the model emerges from considering non-exponentially distributed gonotrophic cycles without any assumption on vector senescence. We will demonstrate that for a general choice of gonotrophic cycle distribution the equilibrium biting rate of the vector population upon hosts is the expected total number of bites made in per vector lifetime divided by the vector life expectancy. This has a concise mathematical expression:

$$E\left[\text{rate of vector population bites per host}\right]=M\mu \frac{\phi_{G}\left(\mu \right)}{1-\phi_{G}\left(\mu \right)}.$$ (7)

Eq (7) is consistent with the classical prediction of biting rating from the vector population in that if the biting process is Poisson (waiting times *G* are exponentially distributed) then,

$$\phi_{G}\left(\mu \right)=\frac{\alpha }{\alpha +\mu }\Rightarrow M\mu \frac{\phi_{G}\left(\mu \right)}{1-\phi_{G}\left(\mu \right)}=M\alpha .$$ (8)

Although eq (7) holds in general, eq (8) is specific to the Poisson assumption.

A second quantity that is key to calculating the basic reproductive ratio is the expected number of infectious vector bites. Infectious bites are those that occur after the latent EIP duration—we denote this quantity *B*_*I*_ (see eq (20) below). In order to make at least one infectious bite an inoculated vector must survive its EIP *and* any remaining gonotrophic period after its EIP finishes. This implies three key considerations:

1. A significant proportion of inoculated vectors (1 − *P*_*E*_; eq (5)) will die before the end of their EIP and contribute no infectious bites.
2. Inoculated vectors that survive their EIP might still die before they complete their remaining gonotrophic period between the end of the EIP and the vector’s first infectious bite.
3. The remaining period will be typically shorter than a complete gonotrophic cycle. Therefore, an infectious vector that survives its EIP will, on average, achieve more infectious bites than a typical emerging adult vector.

Because a vector’s inoculating bite also initiates its EIP it is possibly to calculate *B*_*I*_ exactly using an integral formula [eq (20)]. We can also approximate *B*_*I*_ explicitly, using the asymptotic distribution of remaining time until next bite,

$$E\left[\#\text{ bites after EIP by inoculated vector}\right]=B_{I}\approx \phi_{E}\left(\mu \right)\frac{\alpha }{\mu }.$$ (9)

The approximation is exact if the gonotrophic cycle is exponentially distributed.

Putting Eqs (7) and (9) together gives the generalised reproductive ratio:

$$R_{0}^{g}=\frac{MV}{\gamma }\frac{\mu \phi_{G}\left(\mu \right)}{1-\phi_{G}\left(\mu \right)}B_{I},$$ (10)

and the approximate generalised reproductive ratio (${\tilde{R}}_{0}^{g}$):

$${\tilde{R}}_{0}^{g}=\frac{MV\alpha }{\gamma }\frac{\phi_{E}\left(\mu \right)\phi_{G}\left(\mu \right)}{1-\phi_{G}\left(\mu \right)}.$$ (11)

Eq (11) explains the discrepancy in the introduction. If the EIP duration is fixed at *E* = 1/*σ* then *ϕ*_*E*_(*μ*) = exp{−*μ*/*σ*} which recovers the classical $R_{0}^{c}$ [eq (1)] whereas assuming an exponentially distributed EIP (*ϕ*_*E*_(*μ*) = *σ*/(*σ* + *μ*)) recovers the alternative *R*_0_ [eq (2)]. A key point is that the generalised predictions for the reproductive number $R_{0}^{g}$ depends on the MGFs of the EIP and gonotrophic cycle. MGFs depend upon the full distribution, therefore $R_{0}^{g}$ can be estimated using all the information from an entomological study rather than just summary statistics such as mean (or variance) of gonotrophic cycle duration. In Results we will concentrate on gamma distributed durations which are determined by their mean and dispersion around the mean, however there is no theoretical necessity to use these distributions in general.

### Vector life duration and survival probabilities

Each vector has an age *a*, which defines the time since its first gonotrophic cycle was initiated after the end of its juvenile period. After reaching adulthood the vector has a random exponentially distributed lifetime *L* with mean E[L]=1/μ described by the probability density function,

$$f_{L}\left(a\right)=\mu \exp\left\{-\mu a\right\}.$$ (12)

Exponentially distributed lifetimes, which derive from a constant hazard, have the advantage of a memoryless property. Having survived to some age *a* the vector’s expected remaining life is still 1/*μ*; that is the age of vector doesn’t effect its hazard of dying. Since we acknowledge that the age of the vector effects its biting rate we necessarily consider the age distribution of the vector population. In principle the vector age distribution could be seasonally varying, however we focus on the age distribution at population equilibrium. The age of a vector selected at random from its population at equilibrium is a random variable *A*. The equilibrium age probability density (*f*_*A*_(*a*)) can be calculated using the *microcosm principle* for population processes [20] which states that the proportion of a population in some state is proportional to the mean lifetime an individual would spend in that state. In this case, the microcosm principle gives,

$$f_{A}\left(a\right)=\frac{P(L>a)}{E[L]}=\mu \exp\left\{-\mu a\right\}.$$ (13)

For exponentially distributed life-times, the equilibrium population density of vector age is equal to the life duration density i.e. *f*_*L*_ = *f*_*A*_, but this is not generally true.

After successful inoculation with the pathogen the vector will become infectious after a random EIP duration *E* with density function *f*_*E*_. The memoryless property of the vector lifetime distribution implies that the probability of surviving its EIP (*P*_*E*_) is given by the MGF of the EIP duration evaluated at the vector mortality rate:

$$\begin{array}{ll} P_{E}=\mathbb{P}(L>E) & =\int_{0}^{\infty }\int_{0}^{\infty }1(x>y)\mu \text{ exp}\{-\mu x\}f_{E}(y)\text{ d}x\text{d}y\\ & =\int_{0}^{\infty }\text{exp}\{-\mu y\}f_{E}(y)\text{ d}y\\ & =\phi_{E}(\mu ). \end{array}$$ (14)

Where 1(.) is an indicator function, taking the value 1 if the statement is true or 0 otherwise. Since exp{−*μx*} is a convex function in *x* then Jensen’s inequality gives that for *any* distribution of *E* (c.f. inequality (2.23) in Klebaner [21]):

$$P_{E}\geq \exp\left\{-\mu E\left[E\right]\right\}=\exp\left\{-\mu /\sigma \right\}.$$ (15)

Eq (15) shows that the probability of an inoculated vector surviving a fixed EIP is the lowest possible estimate for any EIP distribution.

### Vector biting process

Vector biting follows a natural repeating cycle: the vector finds a host to bite at the end of each of its gonotrophic cycles and, once successfully fed, initiates a new gonotrophic cycle which includes locating a suitable oviposition site, oviposition, oogenesis and then subsequent host seeking. We model the gonotrophic cycle durations as a collection of independent and identically distributed (i.i.d) random variables labelled *G*_*n*_, each with a common density function, *f*_*G*_. The (random) number of bites the vector has managed by age *a*, denoted *B*(*a*), is the same as the number of gonotrophic cycles completed,

$$B\left(a\right)=\max\left\{n:\sum_{k=1}^{n}G_{k}\leq a\right\}.$$ (16)

Processes such as this that count the number of i.i.d. waiting periods completed up to a given time are known as *renewal processes*, and their probabilistic properties have been extensively analysed [15]. For vectors dying at constant rate *μ*, the chance of surviving each gonotrophic cycle is independent of the number of cycles survived in the past. Eq (14) gives the probability of a vector surviving its EIP, by exactly the same argument the vector survives each gonotrophic cycle with probability *ϕ*_*G*_(*μ*) up until its death therefore the lifetime number of bites by a vector is distributed geometric, with mean *ϕ*_*G*_(*μ*)(1 − *ϕ*_*G*_(*μ*))^−1^.

The Poisson process, which is associated with constant rates and hence ODE models, is an important special case of a renewal process. The implicit assumption that the average biting rate per vector is some fixed rate *α*, independent of the age and time since last bite of the vector, is equivalent to assuming that each gonotrophic cycle is exponentially distribution with average duration E[G]=1/α. However, we do not need to restrict our attention to this special case. Over a long life duration the elementary renewal theorem [15] gives that the expected bites per unit time will converge on the inverse of the average duration *α*,

$$\lim_{a\to \infty }\frac{E[B(a)]}{a}=\alpha .$$ (17)

Eq (17) potentially explains why it is uncommon in the mathematical epidemiology literature to model epidemic contact processes as general renewal processes, as opposed to Poisson processes, since if the life time of an individual is long compared to its inter-contact periods a Poisson process is a reasonable approximation. However, for short lived vectors this is not true; the average biting rate per vector will crucially depend upon the age distribution amongst the population.

We can now write the average biting rate, denoted *α*(*a*), for vectors at age *a* in terms of probability densities:

$$\alpha \left(a\right)=\sum_{n\geq 1}f_{G}^{*n}\left(a\right).$$ (18)

Where $f_{G}^{*n}$ is the density function for the sum of gonotrophic cycles $\sum_{k=1}^{n}G_{k}$ (see supporting information S1 Text). The equilibrium population average biting rate per vector is therefore,

$$\begin{array}{ll} ME[\alpha (A)] & =M\mu \int_{0}^{\infty }\text{exp}\{-\mu a\}\alpha (a)\text{ d}a\\ & =M\mu \underset{n\geq 1}{\sum }{\left[\phi_{G}(\mu )\right]}^{n}\\ & =M\mu \frac{\phi_{G}(\mu )}{1-\phi_{G}(\mu )}. \end{array}$$ (19)

**Fig 2:**
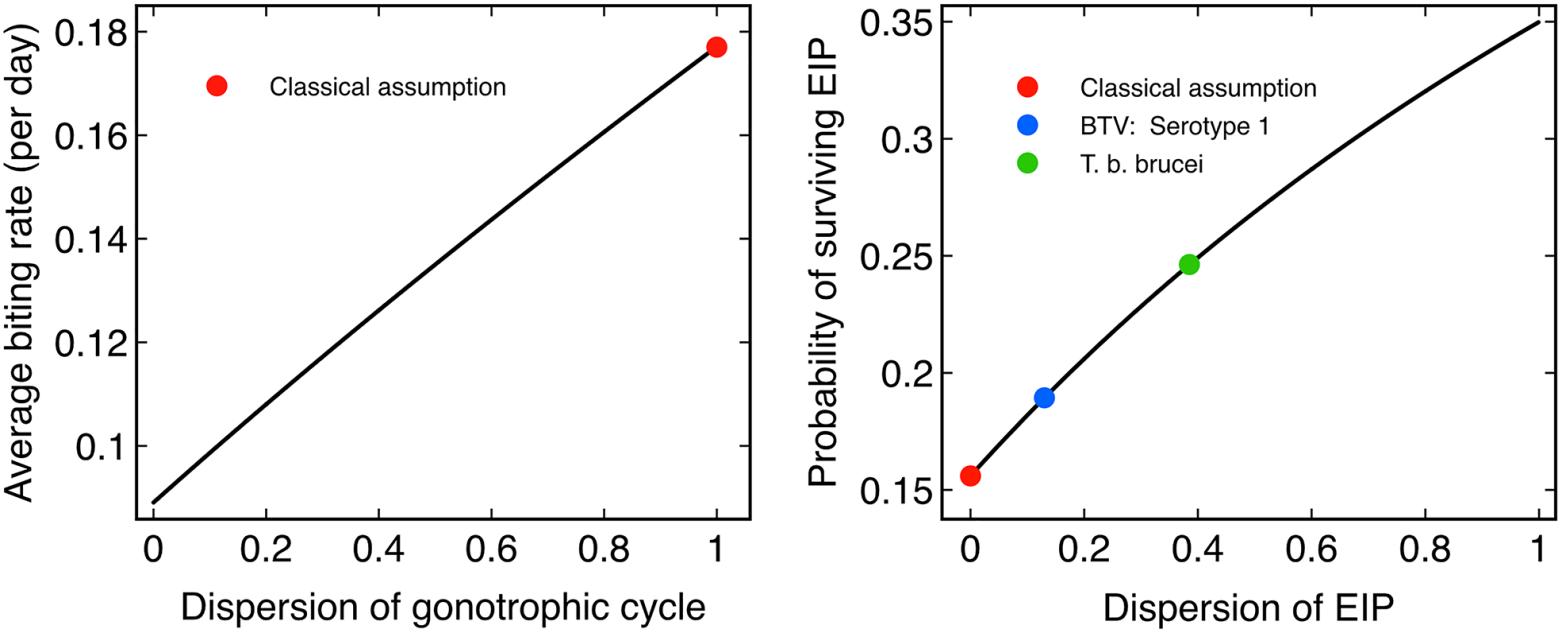
The effect of dispersion on the important epidemiological quantities in VBDs. The equilibrium average biting rate per midge (*left*), the probability of an inoculated midge surviving its EIP (*right*) for *Culicoides* genus midges. The mortality rate is *μ* = 0.22 per day (*Culicoides* genus midge mortality rate at 20°C [13]), the asymptotic biting rate *α* = 0.18 per day (*Culicoides* genus midge inverse mean gonotrophic cycle duration at 20°C [22]), mean EIP *σ*^−1^ = 8.4 days (the posterior median for *Culicoides* genus midge mean EIP duration [19]). The red dots indicate the results derived from the classical Ross-Macdonald assumptions, other colour dots represent EIP dispersion estimates in the literature: BTV-1 spread by *C. bolitinos* midges [19] and *Trypanosoma brucei brucei* spread by tsetse flies [23]. Each quantity is found to be increasing with increasing dispersion.

Here we have used standard results on the MGF of sums of independent random variables and geometric sums. The equilibrium biting rate E[α(A)] differs from the long term biting rate *α* because of the non-exponentially distributed gonotrophic cycles and the typically short lives of the vector, such that many vectors may die before they seek their first blood meal (Fig 2). Note that Eq (19) implies that in the long life limit E[α(A)]→α irrespective of the gonotrophic cycle distribution, and hence results are independent of the assumed distribution.

Vectors that become inoculated via a bloodmeal from an infectious host have bitten at least once, therefore the population equilibrium biting rate is not appropriate to model their subsequent biting process. The expected number of future bites by an inoculated vector *after its EIP*, denoted *B*_*I*_, can be calculated directly as,

$$E\left[\#\text{bites after EIP by inoculated vector}\right]=B_{I}=\int_{0}^{\infty }P\left(L>t\right)P\left(E\leq t\right)\alpha \left(t\right)\text{ d}t.$$ (20)

**Fig 3:**
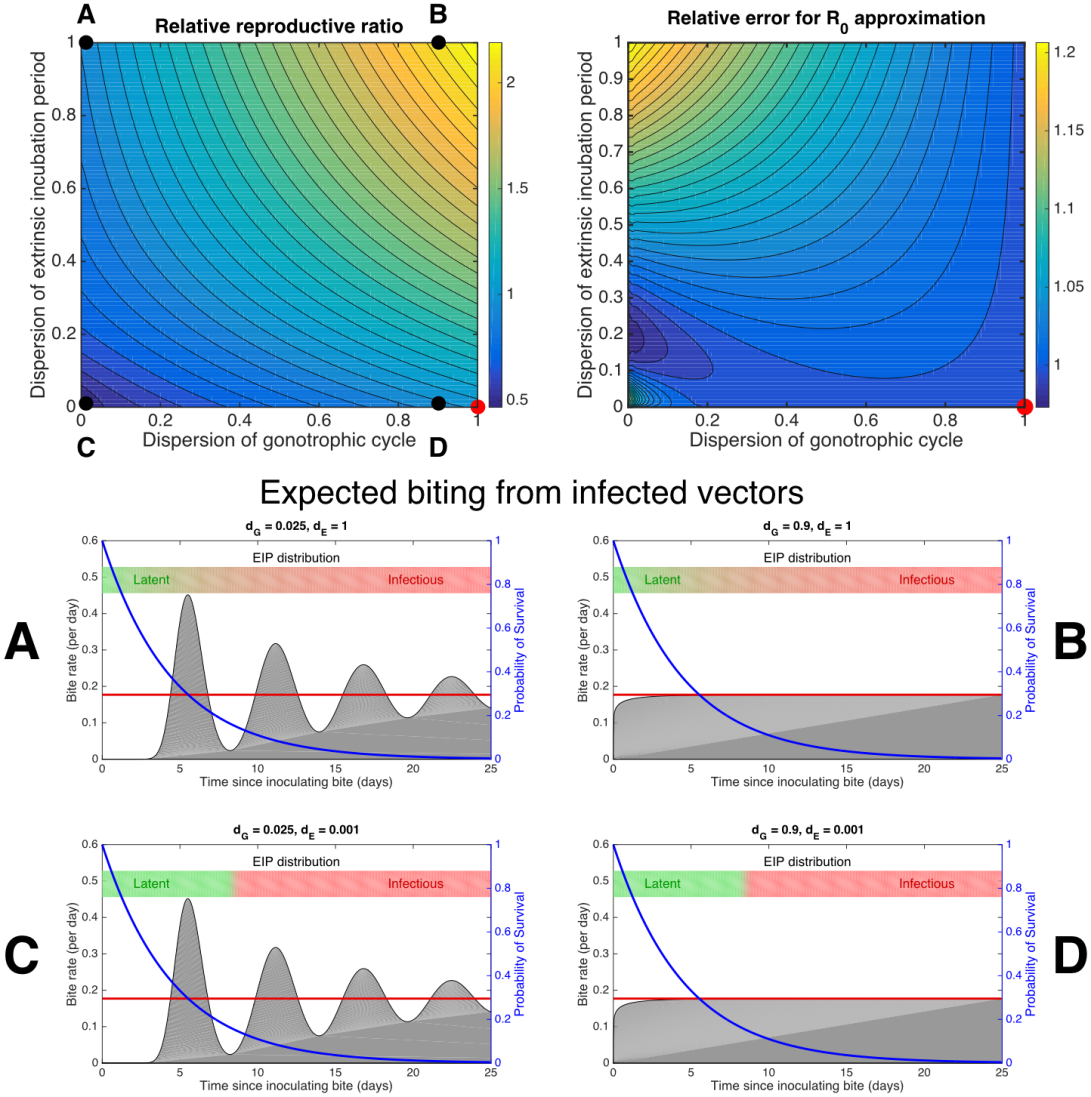
Relative reproductive ratio and expected biting from infected vectors. The generalised reproductive ratio for bluetongue virus relative to its classical estimate $\left(R_{0}^{g}/R_{0}^{c}\right)$ for a range of dispersions (*d*_*G*_, *d*_*E*_) (*top left*) and the relative error of the analytic approximation $({\tilde{R}}_{0}^{g}/R_{0}^{g}$) (*top right*) calculated using the same parameters as in Fig 2 (i.e. for midge life histories assuming a constant 20°C temperature). The lower parameter sets (*middle/bottom, left and right*) give illustrations of the expected life histories of inoculated midges at indicated dispersion parameters. The grey filled curve gives the expected biting rate of midges at each time after the inoculating bite conditional on their survival; the total grey area up to that time is the expected number of bites by the surviving midge. The expected number of actively infectious bites per inoculated midge (*B*_*I*_) is calculated by integrating over the biting rate re-weighted at each time by the survival probability P(L>t) (blue curve; right hand axis) and the incubation probability P(E≤t) (upper bars; green indicating a low probability and red indicating a high probability). The biting rate predicted by modelling bites as a Poisson process (*d*_*G*_ = 1) is shown (red curve). Low dispersion in gonotrophic cycles (parameter sets A,C) predicts that inoculated midge biting is expected to peak around multiples of the average gonotrophic cycle length (*α*^−1^ = 5.5 days) whereas higher dispersion (parameter sets B,D) predicts rapid convergence to the Poisson process biting rate. Low dispersion in EIP predicts a sharp transition at which virtually all inoculated midges will become infectious (parameter sets C,D). In general, higher dispersion favours transmission however the details can be important. The approximation ${\tilde{R}}_{0}^{g}$ is accurate for all values of *d*_*E*_ only when *d*_*G*_ > 0.5, otherwise ${\tilde{R}}_{0}^{g}$ can be either an underestimate or overestimate of $R_{0}^{g}$.

That is the expected infectious bites from an inoculated vector are due to the aggregate of the biting rate at all times *t* after the inoculating bite, weighted by both the probability that the vector is still alive at that time and has completed its EIP (Fig 3). Eq (20) is numerically solvable using that *α*(•) is the rate of change of the solution to the renewal equation with waiting time distribution *G* (see supporting information S1 Text). Probabilistically, the exact value for *B*_*I*_ is difficult to compute because the probability of the vector surviving from the end of its EIP until its next bite depends upon the distribution of *E* in a complex fashion. However, it is clear that only the small proportion of vectors that are by chance comparatively long-lived contribute to *B*_*I*_ when the EIP is typically longer than life expectancy. Therefore it can be argued that the asymptotic biting rate *α* (eq 17) is appropriate for such vectors conditional on having survived their EIP, i.e.

$$B_{I}\approx P_{E}\frac{\alpha }{\mu }=\phi_{E}\left(\mu \right)\frac{\alpha }{\mu }.$$ (21)

Eq (21) is exact when the gonotrophic cycle durations are exponentially distributed. In the supporting information we make the argument for Eq (21) as a general approximation to Eq (20) more rigorous by considering the long-time distribution of remaining time after the EIP until next bite. We also consider the special case of each gonotrophic cycle being of fixed, rather than random, duration (S1 Text).

## Results

We are now in a position to consider the numerical values associated with the quantities formulated above, and consider how they are influenced by the assumed distributions for the length of the gonotrophic cycle and the extinsic incubation period. In particular, we consider the value of the generalised reproductive ratio ($R_{0}^{g}$) using parameters suitable for bluetongue virus spreading between cattle via *Culicoides* genus biting midges. Due to the relative ease by which it can be colonised in a laboratory *C. sonorensis* has become an ideal biological model species for VBDs spread by midges. There exist laboratory and field studies on its mean gonotrophic cycle duration [22, 24], mortality rate [12, 13] and a distributional fit for the EIP of BTV within the midge host [19]. Each of these quantities are sensitive to temperature, so throughout we use the values associated with vector activity at 20°C as illustrative of the theory developed above. See table 1 for a summary of values and functions.

**Table 1:** Parameters and functions for generalised VBD model.

Description	Symbol	Estimate or range	Comments
MGF of random variable X	*ϕ*_*X*_(*θ*)	-	$\phi_{X}\left(\theta \right)=E\left[\text{exp}\left\{-\theta X\right\}\right]$
Mean biting rate at age/time since inoculating bite *a*	*α*(*a*)	-	*a* is used for age since emergence (biting from susceptible vectors), *t* is used for time since inoculating bite (biting from infectious vectors)
Asymptotic biting rate	*α*	0.18 per day	*Culicoides* genus biting midges at 20°C temperature [22, 24]
Vector per capita mortality rate	*μ*	0.22 per day	*Culicoides* genus biting midges at 20°C temperature [13]
Incubation rate of pathogen	*σ*	0.12 per day	*Culicoides* genus biting midges at 20°C temperature [19]
Age of vector chosen at random from population	*A*	0–∞	Density function *f*_*A*_(*a*) = *μ* exp{−*μa*}
Random life span of a vector	*L*	0–∞	Density function *f*_*L*_(*l*) = *μ* exp{−*μl*}
Random duration of gonotrophic cycle	*G*	0–∞	Density function *f*_*G*_, *G* is gamma distributed with mean 1/*α* and dispersion *d*_*G*_.
Random duration of EIP	*E*	0–∞	Density function *f*_*E*_, *E* is gamma distributed with mean 1/*σ* and dispersion *d*_*E*_.
Dispersion of gonotrophic cycle	*d*_*G*_	0–1	$d_{G}=\text{VAR}\left(G\right)/E{\left[G\right]}^{2}$
Dispersion of EIP	*d*_*E*_	0–1	$d_{E}=\text{VAR}\left(E\right)/E{\left[E\right]}^{2}$

In general estimating the reproductive ratio for vector-borne diseases directly from entomological parameters has been problematical, and such estimates often differ strongly from estimates derived from analysing equilibrium inoculation rates (c.f. [25]). In particular, the vector to host ratio *M* is in practise very difficult to estimate. To avoid these issues we present our results in terms of a relative reproductive ratio (*R*_*rel*_), which is the ratio between the generalised reproductive number, $R_{0}^{g}$, and the classical reproductive number, $R_{0}^{c}$, given by Eq (1), as this relative quantity depends only on *μ* and the distributions of *G* and *E*,

$$R_{rel}=\frac{R_{0}^{g}}{R_{0}^{c}}=\frac{\mu^{2}\phi_{G}\left(\mu \right)B_{I}}{\left(1-\phi_{G}\left(\mu \right)\right)\alpha^{2}\exp\left(-\mu /\sigma \right)}.$$ (22)

We note that the classical $R_{0}^{c}$ is an estimate derived by assuming constant biting rates per midge and fixed EIP durations, for example [26]. An additional benefit of considering *R*_*rel*_ is that additional factors such as the contribution due to heterogeneous biting (c.f. [27]) are cancelled out. Determining the distribution of a random period is notoriously difficult so it is convenient to re-express the generalised reproductive ratio in terms of moments of *G* and *E*, and in particular focus on dispersion as a simple measure.

### Dispersion and *R*_0_ for VBDs spread by midges

Using a gamma distribution to interpolate between exponentially distributed (when the gamma shape parameter is 1) and constant periods (when the gamma shape parameter diverges to infinity with fixed gamma mean) is a common modelling choice due to its comparative simplicity and flexibility with a pedigree in epidemic modelling [28]. The dispersion of a gamma distributed random variable *X*, *d*_*X*_, is defined as,

$$d_{X}=\frac{\text{VAR}(X)}{E{\left[X\right]}^{2}}.$$ (23)

The dispersion of the random period *X* interpolates between fixed (*d*_*X*_ = 0) and exponential (*d*_*X*_ = 1), it is also possible for a duration to be over-dispersed (*d*_*X*_ > 1) but we restrict attention to under-dispersal in this work. The dispersion is also usefully incorporated into the MGF of a gamma distributed random variable *X*:

$$\phi_{X}\left(\mu \right)={\left(1+\mu E\left[X\right]d_{X}\right)}^{-1/d_{X}}.$$ (24)

Modelling *E* and *G* as gamma distributed with dispersions, *d*_*E*_ and *d*_*G*_ respectively allows us to calculate the population average biting rate per host and probability of surviving its EIP as

$$ME\left[\alpha \left(A\right)\right]=M\mu {\left({\left(1+\frac{\mu d_{G}}{\alpha }\right)}^{1/d_{G}}-1\right)}^{-1},$$ (25)

and

$$P_{E}={\left(1+\frac{\mu d_{E}}{\sigma }\right)}^{-1/d_{E}}$$ (26)

respectively. The mortality rate, mean gonotrophic cycle duration and mean EIP duration for BTV are all strongly temperature dependent for *C. sonorensis* with the reproductive ratio being maximal at around 20–25°C [9]. At 20°C, using realistic parameters, only a minority of midges are expected to survive the *average* EIP duration of BTV; the chance long-lived tail of the midge population are the sole contributors to the outbreak. However, the actual survival probability for the EIP varies from the 15.6% survival (*d*_*E*_ = 0; the classical assumption that all EIP durations are identical) to 35.0% survival (when *d*_*E*_ = 1). The population averaged biting rate per midge is similarly sensitive, and varies from 0.18 bites per midge per day (*d*_*G*_ = 1; the classical assumption that the gonotrophic cycle is exponentially distributed) to 0.09 bites per midge per day (*d*_*G*_ = 0) (Fig 2).

Turning our attention to the relative reproductive ratio (*R*_*rel*_), and using the numerically exact form of *B*_*I*_ (eq (20) and Fig 3), we find that this increases with increasing dispersion of either distribution. As such, the reproductive ratio is maximised by both distributions being exponential (*R*_*rel*_ = 2.24) and is lowest when both are fixed (*R*_*rel*_ = 0.50) (Fig 3). The classical $R_{0}^{c}$ for BTV can therefore be either a significant overestimate or a significant underestimate compared to the true prediction that accounts for dispersion.

The approximate ${\tilde{R}}_{0}^{g}$ is exact when the midges are expected to bite according to a Poisson process (*d*_*G*_ = 1). Whenever the gonotrophic cycle duration has high dispersal (*d*_*G*_ > 0.5) the approximate reproductive ratio is a good estimate across the entire EIP dispersion range (Fig 3). However ${\tilde{R}}_{0}^{g}$ can be both an underestimate or an overestimate of $R_{0}^{g}$ when *d*_*G*_ < 0.5 depending on an complex interplay between the distributions of *E* and *G*. In particular, when the EIP duration is nearly exponential (*d*_*E*_ ≈1) and the gonotrophic cycle is under-dispersed (*d*_*G*_ < 0.5), with the worst relative error being ${\tilde{R}}_{0}^{g}/R_{0}^{g}=1.21$ when (*d*_*E*_ = 1, *d*_*G*_ = 0). This can be understood through the failure of the assumption that only chance long-lived midges with biting rates well approximated by the asymptotic rate *α* survive their EIP.

### Controlling VBDs spread by midges

The most important feature of the reproductive ratio as a measure of transmission intensity is that it yields a threshold quantity for disease persistence, and hence provides a quantitative insight into control. If an agency tasked with disease control can cause the reproductive ratio to fall below unity for an extended period of time then eradication can be achieved locally. One method for reducing the reproductive ratio is by vaccinating the host population; in the case of BTV there was a mass vaccination of northern European cattle during 2008 which was ultimately effective at eliminating BTV incidence in northern Europe. However, a vaccine for a novel or unexpected strain might not be immediately available, as occurred in northern Europe in 2006 which was invaded by BTV-8 serotype rather than the BTV-2 or BTV-4 serotypes which circulate in southern Europe [17], in which case other control measures need to be considered.

Reducing the midge population by using insecticides would seem to be an obvious solution, if this could be done whilst respecting standards of user and environmental safety. Wide-spread application of insecticide around a farm has not been recorded as successfully causing significant local midge population reduction [29], but it has been suggested that targeting spraying of an insecticide with long residual life in proximity to cattle might be more successful [30]. To model the effect of such insecticide spraying proximate to cattle we assume that the midge mortality is increased by an excess mortality *μ*_*e*_ but the overall midge to host ratio (*M*) remains unchanged, due to the inability to target the entire midge population. If we make the classical assumptions (*d*_*E*_ = 0, *d*_*G*_ = 1), then the critical excess mortality ($\mu_{c}^{*}$) required to achieve eradication can be determined as the solution to:

$$\left(\frac{MV\alpha^{2}}{\gamma (\mu +\mu_{c}^{*})}\right)\exp\left(-\left(\mu +\mu_{c}^{*}\right)/\sigma \right)=1.$$ (27)

This is derived from eq (1), by including the excess mortality and insisting that the resultant reproductive ratio is one.

For disease control using vaccination there is a simple relationship between the critical vaccination coverage of hosts and the reproductive ratio; such that the critical vaccination coverage amongst hosts implied by a classical estimate $R_{0}^{c}$ is $1-1/R_{0}^{c}$. For eradication via increasing midge mortality the relationship between the critical excess mortality and the reproductive ratio estimate $R_{0}^{c}$ derived using the classical assumptions (e.g. [26]) solves,

$$\frac{\mu }{\mu +\mu_{c}^{*}}\exp\left\{-\mu_{c}^{*}/\sigma \right\}=\frac{1}{R_{0}^{c}}.$$ (28)

Note that $\mu_{c}^{*}$ depends on the reproductive ratio estimate $R_{0}^{c}$, the midge mortality rate *μ* and the incubation rate *σ* in contrast to the critical vaccination coverage of hosts which depends only on $R_{0}^{c}$. A natural consideration is the effect that generalising the distribution of the EIP and the gonotrophic cycle durations has upon the predicted critical excess mortality or critical vaccination coverage needed for eradication.

We begin by considering excess mortality and assume that we have access to a classical estimate of the pre-control reproductive ratio $R_{0}^{c}$. In the generalised setting, the midge population biting rate depends on the precise nature of the excess mortality so we consider two separate scenarios:

- *Scenario 1*: The insecticidal spray increases the mortality rate of midges that have successfully fed on cattle, which are assumed to remain nearby these hosts, *and* it also reduces biting from the susceptible midge population by increasing their per capita mortality. This implies that the critical excess mortality ($\mu_{e}^{*}$) is the solution to:
  $$\frac{\left(\mu +\mu_{e}^{*}\right)\phi_{G}\left(\mu +\mu_{e}^{*}\right)}{\alpha (1-\phi_{G}\left(\mu +\mu_{e}^{*}\right))}\times \frac{\mu B_{I}\left(\mu +\mu_{e}^{*}\right)}{\alpha \exp(-\mu /\sigma ))}=\frac{1}{R_{0}^{c}}.$$ (29)
- *Scenario 2*: The insecticidal spray increases the mortality rate of midges that have successfully fed on cattle, which remain nearby these hosts, *but it does not* reduce biting from the susceptible midge population which have yet to be affected by the insecticide. This implies that the critical excess mortality ($\mu_{e}^{*}$) solves the following equation:
  $$\frac{\mu \phi_{G}\left(\mu \right)}{\alpha (1-\phi_{G}\left(\mu \right))}\times \frac{\mu B_{I}\left(\mu +\mu_{e}^{*}\right)}{\alpha \exp(-\mu /\sigma ))}=\frac{1}{R_{0}^{c}}.$$ (30)

Where $B_{I}\left(\mu +\mu_{e}^{*}\right)$ is the expected number of bites after its EIP by a midge undergoing excess mortality $\mu_{e}^{*}$. It should be noted that Eqs (29) and (30) are defined in terms of an estimate of the reproductive ratio based on classical assumptions, which can be related to a generalised reproductive ratio via Eq (22). For either control scenario calculating an approximate critical excess mortality (${\tilde{\mu }}_{e}^{*}$) by using the approximate expression for *B*_*I*_ Eq (21) is significantly easier numerically. The quality of the analytic approximation to the critical excess mortality has similar dependency on the dispersion parameters as the approximate reproductive ratio ${\tilde{R}}_{0}^{g}$: the approximation is broadly good when *d*_*E*_ < 0.5 or when the gonotrophic cycle is more dispersed than the EIP (S1 Fig).

**Fig 4:**
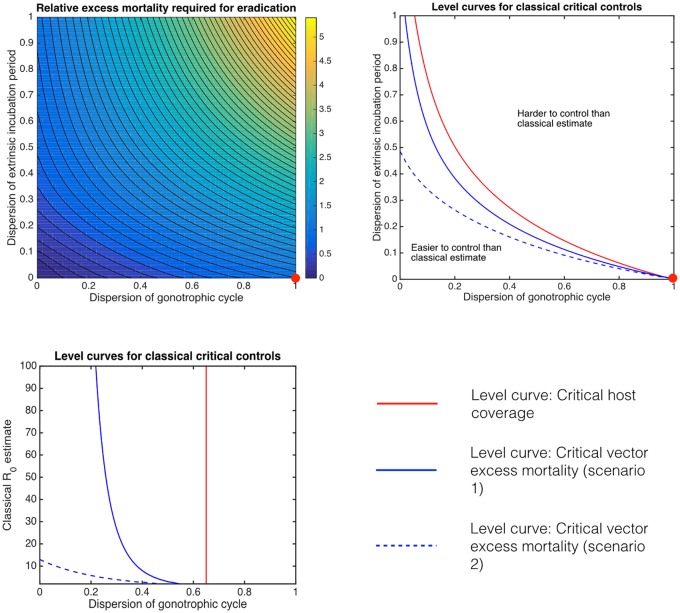
Critical control effort relative to classical predictions. The relative excess mortality that would be required to control BTV using insecticides compared to its classical value $\mu_{rel}^{*}=\mu_{e}^{*}/\mu_{c}^{*}$ given $R_{0}^{c}=2$ (*top left*). The red dot indicates the classical Ross-Macdonald prediction. The environmental temperature is assumed to be a constant 20°C giving the same parameters as in Fig 2. The critical excess mortality required to achieve eradication using insecticides as predicted by classical assumptions can be either an underestimate or an overestimate. Tracing the level curves from the classical value for critical vaccination coverage ($R_{rel}=R_{0}^{g}/R_{0}^{c}=1$; red curves) and critical excess mortality ($\mu_{rel}^{*}=1$; blue curves) separates the (*d*_*G*_, *d*_*E*_) plane into regions where control is more or less difficult compared to the classical value for both cattle vaccination and insecticide spraying. Two scenarios for the efficacy of spraying are considered: where the excess mortality reduces the population biting rate as well as reducing the life expectancy of inoculated vectors (solid blue curves) or if it solely decreases the life expectancy of inoculated vectors (dashed blue curves). For fixed $R_{0}^{c}=2$ (*top right*) and fixed *d*_*E*_ = 0.130 (based on *C. bolitinos* inoculated with BTV-1 serotype [19]; *bottom*) the level curve for *R*_*rel*_ = 1 is distinct from the level curve *μ*_*rel*_ = 1.

The classical formulation of the reproductive ratio Eq (1) suggests that increasing the mortality rate of vectors could be an effective control strategy since $R_{0}^{c}$ decreases faster than exponentially with increasing *μ*. In the generalised setting this is not necessarily true; the sensitivity of $R_{0}^{g}$ to *μ* can be sub-exponential, implying that increasing vector mortality is less effective at reducing the proportion of inoculated vectors expected to survive their EIP than the classical prediction would predict. On the other hand, in the generalised setting the mean vector population biting rate can decrease with increasing *μ*; an effect that reduces transmission and which is absent from the classical Ross-Macdonald model. In fact, the generalised critical value, $\mu_{e}^{*}$, can be either greater or less than the value implied by the classical assumptions ($\mu_{c}^{*}$) derived from solving Eq (28), with the relative critical excess mortality ($\mu_{rel}^{*}=\mu_{e}^{*}/\mu_{c}^{*}$) increasing for both increasing *d*_*G*_ and *d*_*E*_ (Fig 4). Unsurprisingly, the parameter region where control using spraying is expected to be harder than the classical prediction (i.e. $\mu_{rel}^{*}>1)$ is larger for scenario 2, which is more pessimistic about the efficacy of insecticidal spraying than scenario 1 (Fig 4). Because the critical vaccination coverage can be expressed as depending only on the reproductive ratio, the curve *R*_*rel*_ = 1 separates the region where it is more difficult from the region where it is less difficult to eradicate using host vaccination than the classical prediction. However, the *R*_*rel*_ = 1 curve is not identical to the curve defined by $\mu_{rel}^{*}=1$ (Fig 4). This has an important consequence, even if $R_{0}^{g}=R_{0}^{c}$ (i.e. the critical host vaccination coverage is the same even when accounting for the distributions) then the critical amount of excess mortality that insecticide based control must achieve in order to eradicate may be different depending on the distributions. In fact, for *culicoides* biting midges the amount of necessary insecticide control predicted using classical assumptions is an underestimate everywhere along the *R*_*rel*_ = 1 level curve for scenario 1, and the underestimation is even more dramatic for scenario 2. In fact, whenever the generalised reproductive ratio and the classical reproductive ratio coincide, the classical model is always overly optimistic about the how little excess mortality will be required to achieve eradication. This is true for both varying (*d*_*G*_, *d*_*E*_) for a BTV epidemic with the classical estimate $R_{0}^{c}=2$ and when fixing *d*_*E*_ = 0.130 (the median posterior estimated dispersion for the EIP of *C.bolitinos* infected with BTV-1 serotype [19]) allowing $\left(R_{0}^{c},d_{G}\right)$ to vary (Fig 4). However, it should be noted that there is an appreciable region of parameter space where eradication is easier to achieve than expected using classical modelling assumptions. If *d*_*G*_ is sufficiently small then in most cases we predict that it could be easier to eradicate via increasing adult vector mortality than predicted by classical modelling. Whether the findings of this paper are optimistic or pessimistic with regard to insecticidal spray efficacy will ultimately be resolved by more detailed data on biting vector gonotrophic cycle dispersion.

## Discussion

### Modelling mortality associated with vector biting and the first gonotrophic cycle

In the model presented here we assume that vector mortality is due to a constant background hazard rate *μ* and the vectors’ first gonotrophic cycle after emergence as an adult is similar to subsequent cycles. An alternative is to explicitly include heightened mortality risk associated with vector activities such as host-seeking or oviposition as has been done in modelling papers of mosquito-borne diseases (e.g. [31]). Introducing additional risk of vector mortality immediately before and after biting modifies the time-dependent biting rate *α*(•) by incorporating the chance that biting leads to vector death. *α*(•) is the solution to a modified renewal equation and therefore introducing this extra model component represents no extra difficulty compared to only having background mortality (see S1 Text).

Many midge species are autogenous: their females emerge as adults ready to produce and oviposit an initial egg batch without a bloodmeal [32]. From an midge ecology perspective this allows the midge population to persist even when hosts are frequently absent. Therefore, from an epidemiological perspective the waiting time until the first bite from a female autogenous midge on a host occurs at the end of the second gonotrophic cycle. This possibility can be included in our model since we base our analysis on renewal theory: renewal processes with an initial waiting period that is different from subsequent waiting periods (known as delayed renewal processes [15]) are theoretically standard (S1 Text).

We did not explore these biologically motivated modelling extensions further due to our focus on bluetongue diseases spread by *Culicoides* genus biting midges; current best estimates of midge mortality in the wild are based on estimates of the proportion of parous midges in the population [13]. This gives an estimate of the mortality rate based on the probability of surviving a gonotrophic cycle which cannot be disentangled into separate hazards. Moreover, no autogenous midge species has been implicated in the transmission of any arbovirus, including bluetongue [32].

### Concluding remarks

The classical Ross-Macdonald theory of VBDs created a critically important scientific framework with a measure of generational transmission intensity (*R*_0_) at its heart. However the specific practical insights derived from the Macdonald reproductive ratio (for example the implied efficacy of increasing the mortality rate of adult insect vectors in reducing transmission) are undermined by the growing realisation amongst epidemiologists of mosquito-borne diseases that different methods for estimating *R*_0_ produce strongly differing results (*cf.* Smith *et al* for a discussion on the problems associated with validation of the mathematical theory of mosquito-borne pathogens [6]). The problems with theoretic cross-validation appear to have not been explicitly stated in the literature of midge-borne pathogens but the issues are essentially similar. In summary, it has become generally accepted in the modelling community that, whilst all models are simplifications of reality, the classical simplifications of Ross-Macdonald theory may produce misleading results (*cf.* recent reviews [5, 6]). The development of mathematical models of VBDs that deviate from Ross-Macdonald theory has generally focused on heterogeneity in transmission, whether due to heterogenous distribution of vectors across space, for example the recent northern European outbreak of BTV has inspired a number of explicitly spatial models (for example [33–35]), or because of the observation that (typically) a disproportionately greater number of bites will be distributed across a small number of vertebrates (as discussed in [26]). It seems that questions about the role of chance in generating variation in life histories between vectors, even if they are otherwise identical from a modelling perspective, has been largely overlooked despite being in principle empirically observable in the laboratory setting.

In this work, we have demonstrated that relaxing the classical assumptions on the random distributions underlying the calculation of a reproductive ratio for a VBD can have a significant impact on the prediction, even without additional modelling of heterogeneous biting or spatial vector distribution. The impact of the precise distribution of life history events is accentuated for short-lived vectors; thus the economically important vector-disease complex of BTV spread by, the typically short-lived, *culicoides* genus biting midges is an ideal case study for our theory. Our main results are presented in terms of moment generating functions of fairly arbitrary period distributions, so that any reasonable distribution can be used for modelling aspects of the vector life histories. However, our illustrative examples are calculated for the gamma distribution, a natural choice since it interpolates between the constant duration and the exponentially distributed duration. Gamma distributions have been used extensively to model the durations of latency (EIP) and viraemia, most commonly as a discrete sum of exponential distributions (a special case of the gamma distribution called the Erlang distribution), in both the vector-borne disease literature [9] and the directly transmitted disease literature [28]. However, the timing of epidemiological relevant contacts, (*e.g.* successful bites) are overwhelmingly modelled as a Poisson process throughout the mathematical modelling literature. We show that the generalisation of the contact model from a Poisson process to a renewal process, in conjunction with the typically short lives of the vector and a general EIP duration, has significant effects on the basic predictions of an otherwise standard VBD model.

The more general modelling of the vector’s biting process as a renewal process, and the EIP duration as non-constant, predicts that the classical reproductive ratio can be either a significant overestimate or underestimate compared to the generalised prediction. Moreover, the generalised modelling approach cannot be disentangled from considerations of the age distribution of the vector population. However, by assuming that the population is at equilibrium and making theoretically well-motivated approximations (as well as assuming constant mortality rates) we are still able to present the dependence of *R*_0_ on the entomological situation in an explicit manner. For the motivating case of midges spreading BTV, the reproductive ratio is increasing with increasing variation in either the latency (EIP) duration or the time between bites (gonotrophic cycle). This can be understood by considering two consequences of the short life-span of the ‘typical’ midge: firstly, a significant proportion of midges can die whilst their expected biting rate is low (due to relaxing the Poisson biting process assumption) and secondly, that the ‘typical’ midge inoculated with BTV will not survive her EIP (which can depend sensitively on the EIP distribution). Only the ‘tail’ of the inoculated midge population actively contribute to BTV incidence amongst hosts, and therefore random variation between vector life history events becomes a crucial factor. In particular, the potential importance of the precise details about the distribution of the gonotrophic cycle duration seems to be under-recognised in both the theoretical and entomological literature. These findings echo results from at least one other model which generalises the vector biting process [36] and suggests that greater effort must be devoted to understanding and quantifying the detailed vector biology both at the individual and population levels in order to accurately parametrise these kinds of models.

The popularity of *R*_0_ as a metric of transmission intensity is in a large part due to its uncomplicated relationship with disease eradication: if *R*_0_ can be reduced below unity for a sufficient period of time then the disease must be eradicated. The generalised prediction of *R*_0_ presented in this work therefore leads to a reassessment of the efficacy of standard control measures such as the mass vaccination of hosts or the insecticidal spraying against adult vectors. The predicted critical vaccination coverage amongst hosts can still be expressed as only depending on the estimate of the reproductive ratio in the standard fashion, however the critical excess mortality amongst vectors that must be achieved depends on both the estimate of the reproductive ratio and other entomological/epidemiological factors such as basic vector mortality rate and the incubation rate for the pathogen even when making the classical Ross-Macdonald assumptions about the VBD. Predicting the critical excess mortality in the more general setting is further complicated by the necessity to consider the effect that excess mortality has on the age distribution of the vector population. Trials of the ability of insecticidal spraying aimed at adult midges to reduce the local midge population have been pessimistic, leading to speculation that much more must be known about the resting places of adult midges in order to design better spraying protocols [29]. We would add to this discussion further speculation upon which part of the midge population is affected by the additional environmental hazard of a persistent insecticide. Does insecticidal spraying reduce biting from the susceptible mass of the vector population due to shifting their age profile (our scenario 1), or not (our scenario 2)? In either scenario we find that even when the reproductive value predicted by the generalised model agrees with its classical counterpart then the classical model still under-estimates how much excess mortality must be achieved in order to eradicate. None of the arguments above should be read as implying that insecticidal spraying should not be used as a control measure against a vector-borne disease in any circumstance. It might well be the case that a vaccine for hosts might be unavailable or prohibitively expensive whereas a suitable, and safe, insecticide might be readily available and cheap.

An intriguing possibility is to target the older vectors that make up the tail of the age profile using late life acting insecticides (LLAIs). This has been suggested for controlling malaria so that evolutionary pressure on the mosquito population to develop insecticidal resistance is sharply diminished [37]. Modelling the EIP as random rather than fixed always favours vector survival of EIP [eq 15]. This implies that more ‘young’ inoculated vectors will be actively infectious than expected by a model with a fixed EIP; a pessimistic finding with respect to the efficacy of LLAIs whether the LLAI is effective after a delay since exposure or simply preferentially affects older vectors. An advantage of the modelling approach taken in this work is that the action of the LLAI can be explicitly age-dependent or dependent upon real time since exposure. In Read *et al* [37] the action of the LLAI on the vector is modelled as occurring after a number of vector gonotrophic cycles. However, the age of a vector after a number of gonotrophic cycles is uncertain due to variation in the cycle duration. Therefore, experimental data on age-dependent (or time delayed) LLAI efficacy might be difficult to interpret in the context of a model which focuses only on the number gonotrophic cycles a vector survives. Our modelling approach doesn’t suffer from this problem and experimental data on LLAIs could be integrated into our model directly. This would certainly be an interesting direction for future research.

Mathematical models are now commonly used to underpin policy decisions concerning disease control in both developed and low- and middle-income countries. We have used BTV which affects livestock spread by *Culicoides* genus midges as a motivating example. However, the methodology could equally apply to *Leishmania* in humans spread by sandflies, which have similar entomological characteristics to midges. Similarly, while mosquitoes do not necessarily match all assumptions within our model, mosquito-borne diseases could be accommodated with only a slight increase in model complexity. Our work has highlighted the sensitivity of model predictions to entomological and epidemiological details. This points to further avenues of applied experimental or observational research: 1) the acquisition of more detailed knowledge concerning the life cycles of potential vectors, in particular going beyond the measurement of simple averages to include variability; 2) studies of the life expectancy and age profiles of vector populations in the wild; 3) a more comprehensive investigations of the response of vector populations to insecticide-based control measures. Developing highly informative policy-relevant prediction for the future control of vector-borne diseases is likely to therefore require a combination of state-of-the-art models, with meticulous quantitative studies of the insect vector.

## Supporting Information

S1 Text Mathematical details on the biting renewal process and the expected number of potentially infectious bites by a vector after its inoculating bite. This gives further results on renewal processes as well as further discussion on the approximate form for the expected number of infectious bites per inoculated vector (*B*_*I*_). Also included is a numerical scheme for computing *B*_*I*_ directly for both when the inter-bite durations are random and fixed.
(PDF)

S1 FigRelative error of the generalised critical excess mortality to its analytic approximation.The classical reproductive ratio estimate given is $R_{0}^{c}=2$. For a large portion of parameter space the critical excess mortality relative to its analytic approximation is reasonably accurate ($\mu_{e}^{*}/{\tilde{\mu }}_{e}^{*}\approx 1$). As with the approximation ${\tilde{R}}_{0}^{g}$, when the EIP distribution is nearly exponential and more dispersed than the gonotrophic cycle the analytic approximation is less successful due to the failure of the assumption that the expected biting rate of vectors which survive their EIP is well represented by their limiting rate *α*. Also, there is a small region around *d*_*E*_ = 0, *d*_*G*_ = 0 where $\mu_{e}^{*}=0$ and ${\tilde{\mu }}_{e}^{*}∼O\left(10^{-3}\right)$.(EPS)
