# Supplementary material for: The Interaction between Vector Life History and Short Vector Life in Vector-Borne Disease Transmission and Control
Source: PLoS Comput Biol. 2016 Apr 29;12(4):e1004837. doi: 10.1371/journal.pcbi.1004837 (PMC4851302; doi:10.1371/journal.pcbi.1004837)
Supplement: S1 Text — This gives further results on renewal processes as well as further discussion on the approximate form for the expected number of infectious bites per inoculated vector (BI). Also included is a numerical scheme for computing BI directly for both when the inter-bite durations are random and fixed. (PDF) [file pcbi.1004837.s001.pdf]

S1 Text: Mathematical details on the biting renewal process and the expected number of potentially infectious bites by a vector after its inoculating bite.

Contents

1 Theory: Laplace transforms, moment generating functions and renewal processes **2**
1.1 Laplace transforms and moment generating functions 2
1.1.1 Gamma distributed durations and survival probabilities. 3
1.2 Renewal processes 3
1.2.1 The renewal function. 4
1.2.2 Remaining time until next event. 5
1.2.3 Delayed renewal processes. 5

2 Application: Modelling vector life histories **6**
2.1 Vector survival probabilities 6
2.2 The biting renewal process 7
2.2.1 Biting from susceptible vectors. 7
2.2.2 Biting from inoculated vectors. 8
2.2.3 Numerical scheme for solving B_I 9
2.3 Approximating the number of infectious bites per inoculated vector 9
2.4 Fixed inter-bite periods 10
2.5 Model extensions 10
2.5.1 Modified first gonotrophic cycles 11
2.5.2 Vector mortality risk associated with biting 11

List of Tables

A Extended table of Parameters and functions. 12

Overview

In this document we give details about the underlying mathematical theory used throughout the main text. Firstly, we give an overview of the relevant background theory on renewal processes, Laplace transforms and moment generating functions (MGFs). Then we give details on how the background theory was applied

to modelling vector life histories in the main text including references to equations in the main text. Wherever possible we keep the same notation as the main text even when discussing the general theory rather than using ‘standard’ notation for renewal process theory (e.g. as used in Grimmett and Stirzaker [15; main text]). Our aim is to immediately make connection between the general theoretical ideas and the specific application on vector modelling.

1 Theory: Laplace transforms, moment generating functions and renewal processes

We describe the underlying theory used in this paper. The results on renewal processes and moment generating functions can be referenced in many standard texts on probability theory (e.g. [15; main text]), the results on Laplace transforms are also standard (e.g. [1]).

1.1 Laplace transforms and moment generating functions

We will require some results for Laplace transforms of functions. We define the Laplace transform of a function g:

$$\hat{g}(\theta) = \int_0^\infty e^{-\theta t} g(t)\,\mathrm{d}t, \tag{1}$$

and the Laplace convolution of two functions g, h,

$$(g * h)(t) = \int_0^t g(s)h(a-s)\,\mathrm{d}s. \tag{2}$$

We use three standard results for Laplace transforms (e.g. [1]):

$$\widehat{g+h} = \hat{g} + \hat{h} \qquad \text{(Linearity)}, \tag{3}$$

$$\widehat{g*h} = \hat{g}\hat{h} \qquad \text{(Convolution to product)}, \tag{4}$$

$$\widehat{\frac{\mathrm{d}}{\mathrm{d}t}g(t)} = \theta\hat{g}(\theta) - g(0) \qquad \text{(Differential rule)}. \tag{5}$$

In this work we will only need to consider positive random variables $X > 0$ which we use to model uncertain durations of time. Each random variable has a distribution specified by their distribution function $F_X(x) = \mathbb{P}(X \leq x)$. If F_X is differentiable then there exists a density function for X which is is the derivative of the distribution function $f_X = F'_X$. The density function can be thought of as the infinitesimal probability density around values the random variable can take, $f_X(x)\,\mathrm{d}t \approx \mathbb{P}(X \in [x, x+\,\mathrm{d}x])$. For each random variable X we can define a moment generating function (MGF) $\phi_X(\theta)$,

$$\phi_X(\theta) = \mathbb{E}[\exp(-\theta X)]. \tag{6}$$

The MGF of many standard random variables can be calculated analytically and the various moments of X can be calculated from the derivatives of ϕ_X. If the positive random variable has a density function f_X we note that the MGF of X is the Laplace transform of its density function,

$$\phi_X(\theta) = \int_0^\infty e^{-\theta x} f_X(x) dx = \hat{f}_X(\theta). \tag{7}$$

1.1.1 Gamma distributed durations and survival probabilities.

In this work we develop theory that applies to any positive duration $X > 0$ however for concreteness we apply the theory to gamma distributions. We parameterise the gamma distribution in a slightly non-standard way, fixing the mean $\mathbb{E}[X]$ and dispersion $d_X = \mathrm{VAR}[X]/(\mathbb{E}[X]^2)$, giving a gamma density function,

$$f_X(x; \mathbb{E}[X], d_X) = \frac{(\mathbb{E}[X]d_X)^{-1/d_X}}{\Gamma(1/d_X)} x^{(1-d_X)/d_X} e^{-x/(\mathbb{E}[X]d_X)}. \tag{8}$$

Where the normalisation constant Γ is the gamma function. The parametrisation we use for the gamma distribution relates to standard parametrisations: the gamma shape parameter is $1/d_X$ and the gamma scale parameter is $\mathbb{E}[X]d_X$.

In this work we fix mean durations to known entomological parameters, but allow the dispersion value to vary since this quantity is generally under-reported or uncertain in the literature. We consider a range of dispersions $d_X \in [0, 1]$, this scales between the exponentially distributed duration with mean $\mathbb{E}[X]$ ($d_X = 1$) and the fixed duration of length $\mathbb{E}[X]$ ($d_X = 0$). To see this: 1) Choosing $d_X = 1$ gives $f_X(x) = \exp\{-x/\mathbb{E}[X]\}/\mathbb{E}[X]$ which is the density function of an exponential random variable with mean $\mathbb{E}[X]$. 2) The MGF for the gamma distribution is:

$$\phi_X(\theta) = (1 + \theta\mathbb{E}[X]d_X)^{-1/d_X}. \tag{9}$$

The limit as $d_X \to 0$ can be found by relating (9) to a standard result,

$$\begin{aligned}
\lim_{d_X \to 0} \phi_X(\theta) &= \lim_{d_X \to 0} (1 + \theta\mathbb{E}[X]d_X)^{-1/d_X} \\
&= \lim_{\epsilon \to \infty} \Big(1 - \Big(\frac{-\theta\mathbb{E}[X]}{\epsilon}\Big)\Big)^{-\epsilon} \\
&= e^{-\theta\mathbb{E}[X]}. \qquad \text{(Standard result)}
\end{aligned} \tag{10}$$

We note that in the limit $d_X \to 0$ the MGF of the gamma distribution duration X becomes the MGF of the constant 'random' duration $X = \mathbb{E}[X]$ i.e. X is fixed at its average with no variation.

In the main text we demonstrate that the probability of a vector surviving for some random duration $X > 0$ whilst undergoing constant mortality at rate μ is the MGF of X evaluated at the vector mortality rate [equation (14; main text)],

$$\mathbb{P}(\text{Vector surviving for random duration } X) = \phi_X(\mu). \tag{11}$$

This is a general result which connects MGFs to survival probabilities.

1.2 Renewal processes

A renewal process $B = (B(t),\ t \geq 0)$ is a stochastic model for representing the number of randomly occurring events that have arrived within each time period $([0, t],\ t \geq 0)$. Events occur instantaneously with a definite arrival time, we denote the arrival time for the nth event T_n. The waiting time between time 0 and

the first event arrival is denoted G_1 and subsequent waiting durations between the $(n-1)^{th}$ and n^{th} event arrivals are denoted G_n. Therefore,

$$T_n = \sum_{k=1}^{n} G_k, \tag{12}$$

and the renewal process is defined at each time in terms of the waiting durations,

$$B(t) = \max\left\{ n \geq 0 \,\middle|\, T_n = \sum_{k=1}^{n} G_k \leq t \right\}. \tag{13}$$

The key underlying assumption that differentiates renewal processes from other event counting processes is that the set of waiting durations $\{G_n\}_{n\geq 1}$ are treated as positive identically and independently (i.i.d.) random variables. Assuming i.i.d. waiting durations implies three properties of renewal processes:

1. Each waiting duration G_n has the same distribution function as a common random variable G with distribution function $F_G(t) = \mathbb{P}(G \leq t)$.

2. Positive waiting durations implies that $F_G(0) = 0$, $f_G(t) = 0$ for $t < 0$ and the density function of $T_n = \sum_{k=1}^{n} G_k$ is

$$f_{T_n} = f_G * f_G * \cdots * f_G = f_G^{*n}. \tag{14}$$

   Where f_G^{*n} is the n-fold Laplace convolution of the density f_G with itself (a simple corollary of theorem 4.8.1 [15; main text]). The distribution function $F_{T_n}(t) = \mathbb{P}(T_n \leq t)$ can be found from integration,

$$F_{T_n}(t) = \int_0^t f_G^{*n}(s) \, \mathrm{d}s. \tag{15}$$

3. The independence of the waiting durations $\{G_n\}_{n\geq 1}$ implies that if we condition on an event arriving at some time T then the duture event arrivals are probabilistically equivalent to a renewal process started at time T. The process is said to *renew* at each event arrival.

1.2.1 The renewal function.

The expected number of events that have arrived by time t is called the renewal function,

$$m(t) = \mathbb{E}[B(t)]. \tag{16}$$

The elementary renewal equation [15; main text, Eq. 3 Section 10.2] gives that $m(t)/t \to 1/\mathbb{E}[G]$ as $t \to \infty$ but we will need finite time results. We will use two different, but equivalent, representations for the renewal function:

- Solution 1: The renewal function m can be solved as an infinite sum [15; main text, Lemma 7 section 10.1],

$$m(t) = \sum_{n \geq 1} F_{T_n}(t). \tag{17}$$

- Solution 2: The renewal function can also be represented as the implicit solution to the integral equation [15; main text Eq. 9 section 10.1]

$$\begin{aligned} m(t) &= F_G(t) + \int_0^t m(t-s) f_G(s) \, \mathrm{d}s \\ &= F_G(t) + m * f_G(t). \end{aligned} \tag{18}$$

The two solutions are identical representations but will be useful in analysing biting from two different sections of the vector population: 1) biting from the susceptible population of vectors and 2) biting from the inoculated vectors.

1.2.2 Remaining time until next event.

At any time t we can define the remaining time until next event as a random variable:

$$R(t) = \Big(\sum_{n=1}^{B(t)+1} G_n \Big) - t. \tag{19}$$

There is an important limit theorem for the distribution function of remaining time until next event [15; main text, Eq. 5 section 10.3]:

$$F_{R(t)}(y) = \mathbb{P}(R(t) \le y) \to F_{R(\infty)}(y) = \frac{\int_0^y [1 - F_G(s)] ds}{\mathbb{E}[G]}, \qquad \text{as } t \to \infty. \tag{20}$$

The limit theorem (20) states that if we pick t a long time after the start of a renewal process $(t \gg 0)$ then the random time remaining until the next event *after* t will be approximately distributed according to the random variable $R(\infty)$. This approximation becomes exact as $t \to \infty$.

1.2.3 Delayed renewal processes.

A common extension to the renewal process model is to assume that the first waiting period has a different distribution to the other waiting durations; that is G_1 is independent of, but not identically distributed to, $(G_n)_{n \ge 2}$ which share the common distribution function F_G. Traditionally, this modelling approach has been used when the first waiting time is expected to be on average longer than subsequent waiting durations and so the extended model is called a *delayed renewal process*. The renewal function for the delayed renewal process is denoted m^d and has analogous solutions to a standard renewal process :

- m^d can be solved as an infinite sum [15; main text, Eq.14 section 10.4],

$$m^d(t) = \sum_{n \ge 1} F^d_{T_n}(t). \tag{21}$$

  Where the delayed distribution functions for the arrival times are:

$$F^d_{T_n}(t) = \int_0^t f_{G_1} * f_G^{*(n-1)}(s) \, \mathrm{d}s. \tag{22}$$

- The delayed renewal function can also be represented as the implicit solution to the integral equation [15; main text, Eq. 12 section 10.4]

$$m^d(t) = F_{G_1}(t) + \int_0^t m^d(t-s) f_G(s)\,\mathrm{d}s. \tag{23}$$

2 Application: Modelling vector life histories

In this section we apply the standard theory presented above to modelling the epidemiologically relevant events in the life of a disease vector: the frequency and timing of biting, the duration of the EIP for inoculated vectors and the life duration. We use the model to calculate two key quantities for R_0 prediction:

- The expected rate of bites per host from the susceptible vector population per host ($M\mathbb{E}[\alpha(A)]$).
- The average number of potentially infectious bites from inoculated vectors on the host population (B_I).

Other variables that effect R_0, namely vector competence, vector-to-host population ratio and mean viraemic duration for hosts have a direct multiplicative effect (Eq. 3; Main text). We make three specific modelling decisions for the vector life histories:

- The bites of a vector after its emergence as an adult are assumed to occur at the arrival times of a renewal process $B(t)$, with the renewal waiting durations being the gonotrophic cycle lengths $(G_n)_{n\geq 0}$. Each gonotrophic cycle duration is modelled as an independent gamma distribution with known mean duration $\mathbb{E}[G] = 1/\alpha$, i.e. the inverse of the asymptotic biting rate, and dispersion d_G. The density function f_G is given by equation (8).
- If a vector is inoculated then only bites after an EIP duration E are potentially infectious. Variation between vectors in EIP duration is modelled by treating $E > 0$ as a gamma distributed random variable with known mean duration $\mathbb{E}[E] = 1/\sigma$, i.e. the inverse of the pathogen development rate, and dispersion d_E, identically distributed for each vector with density function f_E given by equation (8).
- Each vector has a density- and disease- independent mortality rate μ which is equivalent to an exponentially distributed lifespan $L \sim \exp(\mu)$.

2.1 Vector survival probabilities

In order to bite each vector must survive its gonotrophic cycle and potentially infectious bites can only occur after an inoculated vector survives its EIP. The survival probabilities implied by the model can be calculated directly from equations (9) and (11):

$$\mathbb{P}(\text{Vector survives a gonotrophic cycle}) := P_G = \phi_G(\mu) = \left(1 + \mu\frac{d_G}{\alpha}\right)^{-1/d_G}, \tag{24}$$

$$\mathbb{P}(\text{Vector survives its EIP}) := P_E = \phi_E(\mu) = \left(1 + \mu\frac{d_E}{\sigma}\right)^{-1/d_E}. \tag{25}$$

This derives P_E [equation (26; main text)] used in the main text.

2.2 The biting renewal process

We now apply the results from section 1.2 for both biting from the susceptible vector population per host and for biting from inoculated vectors.

2.2.1 Biting from susceptible vectors.

Susceptible adults vectors may or may not have bitten, the only knowledge we have of their biting pattern is that they emerged at some time. Therefore the relevant time for the susceptible vectors biting process is their age a since emergence. The expected rate of biting for a vector at age a is the time derivative of the renewal function (16) which we denote,

$$\alpha(a) = \frac{\mathrm{d}}{\mathrm{d}a} m(a). \tag{26}$$

We use solution 1 for the renewal equation (17) and property 2 of renewal processes,

$$\begin{aligned} \alpha(a) &= \frac{\mathrm{d}}{\mathrm{d}a} \sum_{n \geq 1} F_{T_n}(a) \qquad \text{(Solution 1)} \\ &= \sum_{n \geq 1} f_{T_n}(a) \\ &= \sum_{n \geq 1} f_G^{*n}(a) \qquad \text{(Property 2).} \end{aligned} \tag{27}$$

Equation (27) justifies (equation 18; main text). The expected biting rate from the *entire* vector population will depend on the age distribution of population; that is the age of a vector chosen at random from the adult vector population. We denote this randomly chosen age A with density f_A. At equilibrium the age distribution is $f_A(a) = \mu \exp(-\mu a)$ (equation 13; main text). For vector to host ratio M the expected biting rate from the susceptible population per host is then:

$$M\mathbb{E}[\alpha(A)] = M\mu \frac{\phi_G(\mu)}{1 - \phi_G(\mu)}. \tag{28}$$

Using the argument in equation (19; main text). Applying our specific result for gamma distributed waiting times from section 2.1 to (28) we get:

$$\begin{aligned} M\mathbb{E}[\alpha(A)] &= M\mu \frac{\left(1 + \frac{\mu d_G}{\alpha}\right)^{-1/d_G}}{1 - \left(1 + \frac{\mu d_G}{\alpha}\right)^{-1/d_G}} \\ &= M\mu \left(\left(1 + \frac{\mu d_G}{\alpha}\right)^{1/d_G} - 1 \right)^{-1} \end{aligned} \tag{29}$$

This derives the biting rate per host used in the main text [equation (25; main text)].

2.2.2 Biting from inoculated vectors.

Vectors are inoculated by successful transmission during a bite on an infectious host. Therefore, inoculated vectors must have survived for long enough to make at least one successful bite. The biting process renews at this time (property 3 of renewal processes). Exponentially distributed lifespans have the *memoryless* property, the future life expectancy for the vector after the inoculating bite is still exponentially distributed $\exp(\mu)$. Therefore the relevant time for the future biting of the vector is time since the inoculating bite t, the age of the vector at the inoculating bite is irrelevant to the future chance of biting. We use a numerical technique to solve for the the expected biting rate based on the Laplace transform of solution 2 of the biting renewal equation,

$$\begin{aligned} \hat{m}(\theta) &= \hat{F}_G(\theta) + \widehat{m * f_G}(\theta) \\ &= \hat{F}_G(\theta) + \hat{m}\hat{f}_G(\theta) \qquad \text{(convolution to product)} \\ \implies \hat{m}(\theta) &= \frac{\hat{F}_G(\theta)}{1-\hat{f}_G(\theta)}. \end{aligned} \tag{30}$$

The expected biting rate at time t after the inoculating bite is defined as $\alpha(t) = \mathrm{d}/\mathrm{d}t m(t)$ and $m(0) = 0$. We use the Laplace differential rule [Equation (5)] to construct the Laplace solution for the expected biting rate for inoculated vectors,

$$\begin{aligned} \hat{\alpha}(\theta) &= \theta\hat{m}(\theta) - m(0) \qquad \text{(Differential rule)} \\ &= \frac{\theta\hat{F}_G(\theta)}{1-\hat{f}_G(\theta)}. \end{aligned} \tag{31}$$

We solve for the age varying biting rate $\alpha(a)$ along a pre-defined mesh of a values by using the Talbot numerical Laplace inversion algorithm[1] [2]. The random number of infectious bites over the remaining lifetime of the vector are those which occur *after* a random latency period E *but before* the death of the vector L. This leads directly to a stochastic integral expression,

$$\#\text{ bites after EIP by inoculated vector } = \int_0^\infty \mathbb{1}(L > t)\mathbb{1}(E \le t)\,\mathrm{d}B(t). \tag{32}$$

See Klebaner [21; main text] for further details on stochastic integrals with respect to jump processes. Because we are modelling the life duration, gonotrophic cycle duration and latency period as independent we have,

$$\begin{aligned} \mathbb{E}[\#\text{ bites after EIP by inoculated vector }] = B_I &= \mathbb{E}\Big[\int_0^\infty \mathbb{1}(L > t)\mathbb{1}(E \le t)\,\mathrm{d}B(t)\Big] \\ &= \int_0^\infty \mathbb{P}(L > t)\mathbb{P}(E \le t)\,\mathrm{d}m(t) \\ &= \int_0^\infty \mathbb{P}(L > t)\mathbb{P}(E \le t)\alpha(t)\,\mathrm{d}t. \end{aligned} \tag{33}$$

Equation (33) justifies equation (20; main text) in the main text. We solve equation (33) numerically thus:

[1]Implementation code for MATLAB at http://uk.mathworks.com/matlabcentral/fileexchange/39035-numerical-inverse-laplace-transform.

2.2.3 Numerical scheme for solving B_I

1. Choose a mesh of t values of size $N+1$: $\{0 = t_0 < t_1 < \cdots < t_N\}$. t_N is chosen sufficiently large that $\mathbb{P}(L > t_N) \approx 0$.

2. Use the Talbot Laplace numerical inversion algorithm on equation (31) to solve: $\{\alpha(a_i)\}_{i=0}^{N}$. Directly calculate $\{\mathbb{P}(L > t_i), \mathbb{P}(E \leq t_i\}_{i=0}^{N}$.

3. Use a quadrature rule on the mesh points to numerically evaluate (33).

The computational complexity of this method compares favourable to resolving solution 1 of the renewal equation (17) directly on a mesh of t values, and is accurate for dispersion values $d_G \geq 0.01$. For nearly fixed gonotrophic cycle durations ($0 < d_G < 0.01$) the numerical solution becomes unstable; solving for $\alpha(t)$ can be achieved by solving (33) directly, albeit at greater numerical cost than using the Talbot inversion algorithm (see section 2.4).

2.3 Approximating the number of infectious bites per inoculated vector

Equation (33) gives an exact but implicit result for the expected number of infectious bites made by an inoculated vector. However, it is often convenient to give an explicit, but approximate, result. Each vector has an independent probability $\phi_G(\mu)$ of surviving each gonotrophic cycle to successfully bite again, which implies that the lifetime number of bites by the vector is geometric distributed with mean $\phi_G(\mu)/(1-\phi_G(\mu))$. Because of the renewal property (property 3 of renewal processes), and because the vector life duration is expected to be memoryless, inoculated vectors that survive 1) their EIP E and then 2) the time remaining before its next bite $R(E)$ will make their first bite and then are, probabilistically, equivalent to a vector starting its adult life infectious. Therefore, we can write B_I (33) in probabilistic form,

$$B_I = \phi_{E+R(E)}(\mu)\Big(1 + \frac{\phi_G(\mu)}{1-\phi_G(\mu)}\Big) \quad (34)$$

Where $\phi_{E+R(E)}(\mu)$ is the probability that the vector survives for the duration $E+R(E)$ with constant mortality rate μ [equation (11)]. The random duration $E+R(E)$ is not analytically accessible in general. However if the EIP duration is typically long compared to the convergence rate of expression (20) then we can approximate $E+R(E) \approx E+R(\infty)$ where $R(\infty)$ is distributed, independently of E, according to the limiting distribution which can be determined using equation (20). In this case the limiting density function for the remaining time left in a gonotrophic cycle when choosing a time in the future is

$$\begin{aligned} f_{R(\infty)}(t) &= \frac{\mathrm{d}}{\mathrm{d}t}\alpha \int_0^t [1 - F_G(s)]\,\mathrm{d}s \\ &= \alpha[1 - F_G(t)]. \end{aligned} \quad (35)$$

And we can make an approximation (which limits to being exact) for the probability of the vector surviving to make its first infectious bite,

$$\mathbb{P}(\text{Vector survives EIP and remaining gonotrophic cycle }) = \phi_{E+R(E)}(\mu) \approx \phi_E(\mu)\phi_{R(\infty)}(\mu). \quad (36)$$

We use equation (7) and invert the Laplace differential rule [i.e. $\hat{F}_X(\theta) = \hat{f}_X(\theta)/\theta$], in order to calculate the probability of a vector surviving the limiting random remaining gonotrophic cycle duration,

$$\begin{aligned}
\phi_{R(\infty)}(\mu) &= \hat{f}_{R(\infty)}(\mu) \qquad \text{(Equation 7)} \\
&= \alpha\Big(\int_0^\infty e^{-\mu t}(1 - F_G(t))\,\mathrm{d}t\Big) \\
&= \alpha(1/\mu - \hat{F}_G(\mu)) \\
&= \frac{\alpha}{\mu}\Big(1 - \hat{f}_G(\mu)\Big) \qquad \text{(Inverse differential rule)} \\
&= \frac{\alpha}{\mu}\Big(1 - \phi_G(\mu)\Big) \qquad \text{(Equation 7)}.
\end{aligned} \tag{37}$$

This leads directly to the approximation for B_I used in the main text,

$$\begin{aligned}
B_I &\approx \phi_E(\mu)\phi_{R(\infty)}(\mu)\Big(1 + \frac{\phi_G(\mu)}{1 - \phi_G(\mu)}\Big) \\
&= \phi_E(\mu)\frac{\alpha}{\mu}\Big(1 - \phi_G(\mu)\Big)\Big(1 + \frac{\phi_G(\mu)}{1 - \phi_G(\mu)}\Big) \qquad \text{(Equation 37)} \\
&= \phi_E(\mu)\frac{\alpha}{\mu}.
\end{aligned} \tag{38}$$

This justifies equation (21; main text). The quality of the approximation for B_I depends on how rapidly the remaining time until next bite distribution converges onto its asymptotic distribution, hence the approximation is exact when for exponentially distributed gonotrophic cycles (this can be seen by inspection of (35)). On the other hand, if the EIP duration is more dispersed than the gonotrophic cycle duration, which in turn is significantly under-dispersed compared to the exponential distribution, then the fundamental assumption that the EIP duration is long compared to the convergence rate is poor. This explains why the approximation to B_I is found to be reasonable except in the parameter regime $\{d_G < 0.5,\ d_E > 0.5\}$.

2.4 Fixed inter-bite periods

The only example in the text where the above analysis is inappropriate is when the dispersion of the gonotrophic cycle is exactly zero, $d_G = 0$, which corresponds to the time between successful bites being some fixed value T so that $G_n = T$ for all $n \geq 0$. This transforms the solution to (33) into

$$B_I = \sum_{n \geq 1} \mathbb{P}(L > nT)\mathbb{P}(E \leq nT). \tag{39}$$

Truncating the sum (39) at some value n^* gives an error which is less than $\exp\{-n^*\mu T\}/(1 - \exp\{-\mu T\})$, the error can be chosen arbitrarily small by an *a priori* choice of n^*.

2.5 Model extensions

We discuss two modelling extensions that can be easily added to the general modelling framework presented in this work. Firstly, that the first gonotrophic cycle might be intrinsically different from subsequent gonotrophic cycles and secondly, that as well as a constant mortality rate for vectors there might also be a specific risk associated with biting (both before and after an attempt).

2.5.1 Modified first gonotrophic cycles

It might be more realistic to model the first gonotrophic cycle separately to subsequent gonotrophic cycles. Some midge species are autogenous; that is they lay one batch of eggs before they seek a host. Essentially autogenous midge species go through two gonotrophic cycles before their first bite. The theory of delayed renewal processes (section 1.2.3) is ideal for analysing the consequences of this model modification. We assume that we can gather data on the distribution of the first gonotrophic cycle thus giving an estimate for its density function f_{G_1}. The modified first gonotrophic cycle alters the biting rate from the susceptible population because the probability of vectors surviving to their first bite is altered.

We repeat the analysis of section 2.2.1 using a solution to the *delayed* renewal equation [equation (21)]. This gives the delayed age-dependent biting rate as,

$$\alpha^d(a) = f_{G_1}(a) + \sum_{n\geq 1} f_{G_1} * f_G^{*n}(a). \tag{40}$$

The modified biting rate from the population is therefore,

$$\begin{aligned} M\mathbb{E}[\alpha^d(A)] &= M\mu\phi_{G_1}(\mu)\Big(1 + \frac{\phi_G(\mu)}{1+\phi_G(\mu)}\Big) \\ &= M\mu\frac{\phi_{G_1}(\mu)}{1+\phi_G(\mu)}. \end{aligned} \tag{41}$$

Inoculated vector have, by definition, succeeded in biting at least once so their future biting should still be modelled using a standard renewal process.

2.5.2 Vector mortality risk associated with biting

Constant mortality is an appropriate model for background vector risk such as from predation. However, biting brings its own specific additional risks which we have not modelled. For example, we could model the effect of insecticide deployment near hosts as introducing a risk of vector death both immediately before and immediately after biting. We define p_1 as the probability of the vector surviving the risk before biting and p_2 as the probability of the vector surviving the risk after biting. We denote the modified renewal equation for inoculated vectors m^r. The probability that the vector survives to make its next bite after inoculation p_1p_2, we condition on this happening and the timing of the next biting to give,

$$\begin{aligned} m^r(t) &= p_1p_2\Big(\int_0^\infty \mathbb{E}[B(t)|T_1 = s]f_G(s)\,\mathrm{d}s\Big) \qquad \text{(Law of total expectation)} \\ &= p_1p_2\Big(\int_0^t \{1+\mathbb{E}[B(t-s)]\}f_G(s)\,\mathrm{d}s\Big) \qquad \text{(Renewal property 3)} \\ &= p_1p_2\Big(\int_0^t f_G(s)\,\mathrm{d}s + \int_0^t m^r(t-s)f_G(s)\,\mathrm{d}s\Big) \\ &= p_1p_2\Big(F_G(t) + m^r * f_G(t)\Big) \end{aligned} \tag{42}$$

The renewal equation (42) can then be used solve for a modified B_I as in section 2.2.2. If we assume that it is only the subsection of vectors that bit on the protected hosts that undergo this extra risk (as in control

scenario 2 in the main text) then the biting from the susceptible population is unaffected. However, this may not be the case. Evaluating the effect of biting risk on the age-distribution of the population is possible and will be a future direction of research.

Table A: Extended table of Parameters and functions.

**Description**	**Symbol**	**Comments/Definition**
Laplace transform of function g	$\hat{g}(\theta)$	$\hat{g}(\theta) = \int_0^\infty \exp\{-\theta t\} g(t)\,\mathrm{d}t$
MGF of random variable X	$\phi_X(\theta)$	$\phi_X(\theta) = \mathbb{E}[\exp\{-\theta X\}]$
Distribution function of random variable X	F_X(t)	$F_X(t) = \mathbb{P}(X \leq t)$
Distribution function of random variable X	$f_X(t)$	$f_X(t) = (\,\mathrm{d}/\,\mathrm{d}t) F_X(t)$
Probability vector survives random period X with mortality rate μ	P_X	$P_X = \phi_X(\mu)$
Biting renewal process for a vector	$B(t)$	# bites in period $[0, t]$
n^{th} gonotrophic cycle for a vector	G_n	$(G_n)_{n\geq 1}$ are i.i.d random variables G is gamma distributed with mean $1/\alpha$ and dispersion d_G.
Asymptotic biting rate	α	Parameter. $\alpha = 1/\mathbb{E}[G]$
Dispersion of gonotrophic cycle	d_G	$d_G = \mathrm{VAR}(G)/\mathbb{E}[G]^2$,
Time n^{th} bite occurs	T_n	$T_n = \sum_{k=1}^{n} G_k$
Renewal equation	$m(a)$	$\mathbb{E}[B(a)]$. a is used for age of vector (modelling bites from susceptible vectors), t is used for time since inoculating bite (modelling infectious vectors).
Mean biting rate at age/time since inoculating bite a	$\alpha(a)$	$\alpha(a) = (\,\mathrm{d}/\,\mathrm{d}a) m(a)$
Remaining time until next bite after time t	$R(t)$	$R(t) = (\sum_{n=1}^{B(t)+1} G_n) - t$
Limiting random time left until next bite	$R(\infty)$	$\lim_{t\to\infty} F_{R(t)}(y) = F_{R(\infty)}(y) = \alpha \int_0^y [1 - F_G(s)\,\mathrm{d}s$
Age of vector chosen at random from population	A	Density function at population equilibrium $f_A(a) = \mu \exp\{-\mu a\}$
Random life span of a vector	L	Density function $f_L(l) = \mu \exp\{-\mu l\}$
Vector per capita mortality rate	μ	Parameter. $\mu = 1/\mathbb{E}[L]$
Random duration of EIP for an inoculated vector	E	E is gamma distributed with mean $1/\sigma$ and dispersion d_E.
Incubation rate of pathogen	σ	Parameter. $\sigma = 1/\mathbb{E}[E]$
Dispersion of EIP	d_E	$d_E = \mathrm{VAR}(E)/\mathbb{E}[E]^2$,
Expected number of infectious bites from an inoculated vector	B_I	Average number of bites after EIP
Delayed renewal equation	m^d	Renewal equation for biting with G_1 with different distribution from other gonotrophic cycles.
Modified renewal equation	m^r	Renewal equation for biting with p_1 prob. of vector before bite and p_2 prob. of dying after bite.

References

[1] LePage, W R. Complex variables and the Laplace transform for engineers. New York: Dover Publications; 1980.

[2] Talbot A. The accurate numerical inversion of Laplace transforms. IMA Journal of Applied Mathematics 23(1) 1979: 97-120.
